# Mixed Type Histology as a Predictive Factor for Esophagojejunostomy Leak in Advanced Gastric Cancer

**DOI:** 10.3390/cancers12061701

**Published:** 2020-06-26

**Authors:** Karol Rawicz-Pruszyński, Katarzyna Sędłak, Radosław Mlak, Jerzy Mielko, Wojciech P. Polkowski

**Affiliations:** 1 Department of Surgical Oncology, Medical University of Lublin, Radziwiłłowska 13 St., 20-080 Lublin, Poland; sedlak.katarz@gmail.com (K.S.); jerzy.mielko@umlub.pl (J.M.); wojciech.polkowski@umlub.pl (W.P.P.); 2 Department of Human Physiology, Medical University of Lublin, Radziwiłłowska 11 St., 20-080 Lublin, Poland; radoslaw.mlak@gmail.com

**Keywords:** advanced gastric cancer, esophagojejunostomy, postoperative complications

## Abstract

Since esophagojejunostomy leak (EJL) after gastrectomy is a potentially fatal complication and may impact the survival of patients with advanced gastric cancer (GC), it is important to establish risk factors for the EJL and to prevent this surgical complication. The aim of this study was analysis of predictors for the postoperative clinically apparent EJL. All patients operated for advanced GC between October 2016 and December 2019 were analyzed from a prospectively maintained database. The evaluation of the EJL and postoperative complications according to the demographic and clinical (categorized) variables was performed with odds ratio test (multivariate analysis was performed with the use of logistic regression method). Among the 114 patients included in the study, 71.1% received neoadjuvant chemotherapy and 19.3% underwent gastrectomy followed by the hyperthermic intraperitoneal chemotherapy (HIPEC). Postoperative EJL was found in 4.6% patients. The risk of EJL was significantly higher for mixed-type GC (OR = 12.45, 95% CI: 1.03–150.10; *p* = 0.0472). The risk of other postoperative complications was significantly higher in patients undergoing HIPEC (OR = 3.88, 95% CI: 1.40–10.80, *p* = 0.0094). The number of lymph nodes removed (>38) was characterized by 80% sensitivity and 79.6% specificity in predicting EJL (AUC = 0.80, 95% CI: 0.72–0.87; *p* < 0.0001). Mixed histological type of GC is a tumor-related risk factor for the EJL. HIPEC was confirmed to be a risk factor for postoperative complications after gastrectomy.

## 1. Introduction

Gastric cancer (GC) is the fifth most frequently diagnosed cancer, with over 1,000,000 new cases and 783,000 deaths in 2018, which makes it the third leading cause of cancer death worldwide [1]. The preferred treatment for advanced, non-metastatic GC is (total) gastrectomy with D2 lymph node dissection. According to the Japanese Gastric Cancer Association, patients with early tumors excluded from endoscopic treatment (cT1N0), can undergo organ-sparing surgery, such as pylorus-preserving gastrectomy and proximal gastrectomy [2]. The type of resection depends mainly on a topographical subsite of GC. In most Asian countries, non-cardia (distal) GC occurs more frequently than cardia (proximal) GC. However, in some Western populations with GC incidence rates lower than the global average, cardia (proximal) GC rates are similar or even higher than distal GC, particularly in men (male-to-female ratio 3:1) [3]. The Laurén classification of GC is widely used in clinical practice, since it reflects GC morphology, epidemiology, tumor biology, clinical management and outcome [4].

In Western countries, the standard of care in advanced GC includes perioperative chemotherapy and gastrectomy with D2 lymphadenectomy [5,6]. European studies have shown that advanced GC patients benefit from neoadjuvant chemotherapy compared to those who receive only surgical treatment [7,8]. Moreover, the use of neoadjuvant chemotherapy does not increase the risk of postoperative complications [9,10]. Conversion systemic therapy is increasingly used in an oligometastatic setting. If regression is achieved, extended gastrectomy followed by hyperthermic intraperitoneal chemotherapy (HIPEC) is amenable for strictly selected patients with limited peritoneal involvement (P1) or positive cytology (CY1) [11,12].

The extent of the resection determines the preferred reconstruction method [13]. Among numerous restoration techniques, esophagojejunostomy (EJ) is feasible in both, radical (total gastrectomy) and organ-sparing (proximal gastrectomy) gastric surgery. Since for stage IB–III GC radical (total) gastrectomy is recommended, EJ is mostly used as the preferred reconstruction technique [5].

Although the overall rate of complications after total gastrectomy has decreased over the last decade, complications requiring surgical re-intervention remained steady over time [14]. An anastomotic leak, in particular, may significantly increase morbidity and mortality rates [14,15]. Postoperative (in-hospital) death after anastomotic leak following total gastrectomy varies from 19 to 62% [16]. Due to the patient’s poor general, cancer-related condition and technical difficulties of the procedure, esophagojejunostomy leak (EJL) remains a critical postoperative condition in both early and advanced GC. The leak rate varies from 5% to 14% [17], and it has been considered a poor prognostic factor [15,17].

The EJL risk factors are divided into patient-, tumor-, and surgery-related [17,18]. Reported patient-related factors include: age ≥ 65 years, male gender, anaemia, malnourishment, cardiovascular disease, pulmonary insufficiency, advanced diabetes mellitus, chronic renal failure, smoking, obesity, high visceral fat area, use of steroids and localization and size of the tumor [15,17,19,20,21,22,23]. Tumor-related risk factors include pathological stage IV [21,24] and esophageal invasion [22], whereas risk factors related to surgery are: intraoperative technical errors, prolonged operating time, excessive tension on the anastomosis, limited vascular supply, and combined splenectomy [17,19,21,25,26,27]. The aim of this study was the analysis of predictors for the postoperative, clinically apparent EJL.

## 2. Results

The clinicopathological features of the 114 patients included in the study are shown in Table 1.

The intestinal type was the most frequent tumor, followed by diffuse- and mixed-type GC (37%, 34.3% and 28.7% of patients, respectively). There were 42.3% of patients with pT3, 19.8% and 11.7% of patients with pT4a and pT4b tumors, respectively. Lymph node metastases (N1-N3) were present in 59.1% of patients, and 26.3% of patients had distant metastases (cM0/pM1). Neoadjuvant chemotherapy was applied in 71.1% of patients. Gastrectomy with HIPEC procedure was used in 19.3% of patients. Postoperative complications were reported in 40.4% of patients. The EJL was found in 5/114 (4.5%) patients. The average CCI value was equal to 17.4 (26.1). In patients with or without EJL, the median of CCI was 79.5 (58.2–100) and 0 (0–20.9), respectively (*p* < 0.00001). The average time of hospitalization and ICU stay was 12.9 days and 6.9 days, respectively.

### 2.1. EJL Risk Factors

The risk of the EJL was significantly higher for the mixed type compared to other histological types of GC (OR = 12.45, 95% CI: 1.03–150.10; *p* = 0.0472; adjusted). The risk of postoperative complications was significantly higher in patients undergoing HIPEC (OR = 3.88, 95% CI: 1.40–10.80, *p* = 0.0094; adjusted). Univariate and multivariate analyses of the risk of EJL and postoperative complications are presented in Table 2.

### 2.2. Comparisons of CCI Values Depending on the Selected Demographic and Clinical Variables

The CCI was significantly higher in patients with EJL (79.5 vs. 0; *p* < 0.0001) and HIPEC (31.6 vs. 0; *p* = 0.0034). Differences in the median CCI, depending on demographic, clinical, and pathological features, are presented in Table 3.

### 2.3. Comparisons of Selected Demographic and Clinical Variables in Patients with Postoperative Complications

Significantly longer hospitalization time was observed in patients with EJL (29 vs. 11 days; *p* = 0.0023) and postoperative complications (12.5 vs. 10 days; *p* = 0.0004). Similarly, significantly longer ICU stay was observed in patients with EJL (12 vs. 4 days; *p* = 0.0071). A significantly higher number of harvested lymph nodes was observed in patients with EJL (41 vs. 27; *p* = 0.0221). Moreover, the number of lymph nodes removed (>38) was characterized by 80% sensitivity and 79.6% specificity in predicting EJL (AUC = 0.80, 95% CI: 0.72–0.87; *p* < 0.0001).

### 2.4. Correlation between CCI Values and Selected Demographic and Clinical Variables

Positive correlations were observed between the CCI index and: hospitalization time (rho = 0.300, *p* = 0.0017) and ICU stay (rho = 0.550, *p* = 0.0223).

### 2.5. Overall Survival

In the study group, median OS was 20 months (range: 0.5–114 months; death was reported in 53.1% of patients). The presence of pT4a or pT4b was associated with an approximately 2-fold higher risk of shortening the median OS (11 vs. 23 months; HR = 1.74, 95% CI: 0.99–3.07; *p* = 0.0287). The presence of pN1-N3 was associated with an approximately 2.5-fold higher risk of shortening the median OS (11 vs. 63 months; HR = 2.52, 95% CI: 1.17–5.46; *p* = 0.0192; adjusted. The presence of the pM1 was associated with an approximately 4-fold higher risk of shortening the median OS (5 vs. 54 months; HR = 4.16, 95% CI: 1.88–9.21; *p* = 0.0005; adjusted). An increased number of neoadjuvant chemotherapy cycles (≥4) was associated with a significantly higher risk of shortening the median OS (6 vs. 23 months; HR = 2.79, 95% CI: 1.10–7.07; *p* = 0.0316; adjusted). Lymph node metastases (n ≥ 2) were associated with a significantly higher risk of shortening the median OS (10 vs. 54 months; HR = 2.15, 95% CI: 1.22–3.79; *p* = 0.0081; adjusted). LNR ≥ 0.07 was associated with a significantly higher risk of shortening the median OS (9 vs. 54 months; HR = 2.44, 95% CI: 1.38–4,30; *p* = 0.0022; adjusted).

## 3. Discussion

The present study was undertaken to evaluate the risk factors of EJL and morbidity in patients with advanced GC. The Laurén mixed histotype has been found to be a new tumor-related risk factor for EJL. Additionally, patients with EJL had a significantly higher number of harvested lymph nodes. The risk of postoperative complications has been significantly higher in patients undergoing HIPEC.

The influence of the Laurén histological type of GC on the occurrence of EJL or postoperative complications in GC patients has not been investigated so far. Recent studies from the Far East have shown more aggressive clinical behavior and worse survival in the mixed type, but mainly for early GC [28,29]. Even though it seems that the mixed type does not influence the OS when compared to the diffuse type in both early and advanced GC [28,30], mixed type histology has been indicated as a poor prognostic factor [31]. Aggressive biological features of mixed type GC include high Ki-67 proliferation index, and abnormal expression of E-cadherin and vascular endothelial growth factor (VEGF) [32]. Tumor size and depth of infiltration additionally exacerbate the clinical outcome [28]. The present study indicates that mixed type GC was a significant risk factor of the EJL compared to other types. Consequently, it could have affected the risk of more frequent postoperative complications.

As previously mentioned, poor vascular supply has been recognized as a surgery-related risk factor for EJL [17,18]. In total and proximal gastrectomy, both right and left cardiac lymph node stations (no.1 and no.2, respectively) are resected [2]. Since lymphadenectomy with at least 15 perigastric nodes, retrieval could result in improved survival [33], an adequate lymph node harvest should be maintained. On the other hand, extensive lymph node dissection in the cardiac region could presumably lead to poor vascularization of the distal esophagus used for the anastomosis, followed by EJL, as shown in our study.

The use of HIPEC in GC has been investigated in both locoregional and metastatic settings [34,35]. Extended gastrectomy combined with peritonectomy and HIPEC may be beneficial to strictly selected patients with oligometastatic peritoneal GC [36,37]. The ongoing phase III RCT’s in the West will determine the potential survival benefit of HIPEC in advanced GC [38,39]. The present study shows that HIPEC might result in an increased risk of postoperative complications. However, it is not associated with an increased risk of postoperative leak and does not affect OS compared to patients not treated with HIPEC. Chouliaras et al. described that gastrectomy plus HIPEC is associated with significant mortality and morbidity [40]. The study indicated that patients who suffered from an anastomosis leak after HIPEC had a decreased median OS of 1.6 years compared with 3.1 years in the no-leak group. In contrast, Piso et al. reported that anastomosis following total or subtotal gastrectomy with HIPEC is safe when performed in experienced centers [41]. However, the study group included only 11 patients with GC, while esophagojejunostomy was performed in 15 patients. In recently published meta-analyses [42,43], a significantly higher risk for postoperative complications was reported in patients undergoing prophylactic HIPEC for advanced GC without overt peritoneal metastases. The incidence rate of anastomotic leak ranged from 2 to 20% [43], yet was without statistical significance when compared to surgery alone groups.

In our experience, anastomotic leak after gastrectomy plus HIPEC was one of the least common (3%) complications, with median CCI of 42.4, whereas the grade 3–5 complication rate was 47% [36]. In the present study, among 22 patients who underwent gastrectomy with HIPEC, only one (4.5%) suffered from a postoperative leak. In contrast, the multicenter French CYTO-CHIP study showed that anastomotic leak was a common complication (35/162; 21.6%), whereas grade 3–5 complications occurred with similar frequency of 53.7% [37].

There are several limitations to the study. Due to the retrospective nature of the study, it cannot specify causation. Due to the relatively small sample size, subgroup stratification analysis might be biased. Moreover, our study was not designed to analyze the impact of EJL on long-term survival.

## 4. Materials and Methods

**Figure 1:**
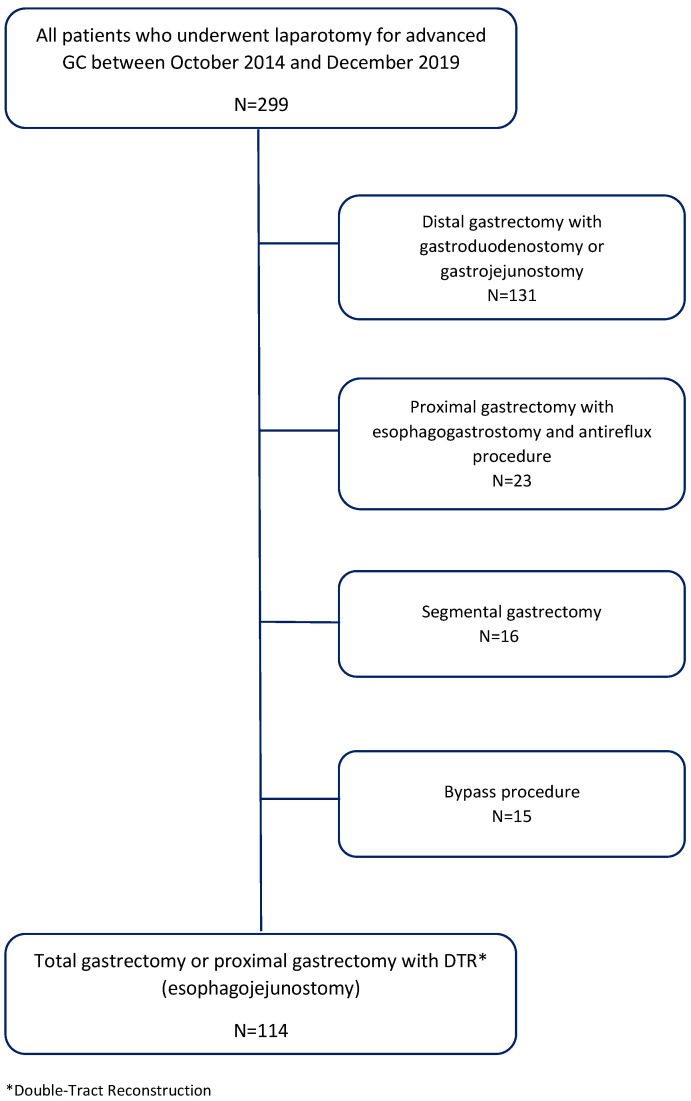
Flow chart of the study.

After having institutional review board approval (Bioethical Committee of Medical University of Lublin, Ethic Code: KE-0254/297/2018), we collected data from a prospectively maintained database of all patients operated on GC between October 2014 and December 2019 in the Department of Surgical Oncology of the Medical University of Lublin (Poland). The inclusion criteria were gastrectomy with direct end to side anastomosis between the stump of the distal esophagus, and jejunum. Thus, patients in whom total gastrectomy followed by Roux-en-Y esophagojejunostomy or proximal gastrectomy with double-tract reconstruction (DTR) were included. The exclusion criteria were: distal gastrectomy followed by Rydygier, Billroth II or Roux-en-Y anastomosis, proximal gastrectomy with esophagogastrostomy and anti-reflux procedure, bypass procedure or segmental gastrectomy. Some 114 patients were eligible for the analysis. A flow chart of the study with numbers of patient selection is presented in Figure 1.

### 4.1. Neoadjuvant Chemotherapy

The perioperative EOX regimen consisted of 50 mg/m^2^ epirubicin and 130 mg/m^2^ oxaliplatin on day 1, with 625 mg/m^2^ capecitabine administered twice daily on days 1–21. The perioperative regimen was repeated 2–3 times every 3 weeks. The FLOT chemotherapy consisted of oxaliplatin, 85 mg/m^2^; leucovorin, 200 mg/m^2^; and docetaxel, 50 mg/m^2^. Each was an intravenous infusion followed by fluorouracil, 2600 mg/m^2^, as a 24-hour continuous intravenous infusion on day 1, repeated every 2 weeks.

### 4.2. Laurén Classification

According to the Laurén histo-prognostic classification, gastric adenocarcinomas are divided into intestinal, diffuse, mixed and indeterminate types [44]. The intestinal type consists of well-formed tubules, whereas the diffuse type is characterized by tumor cells that show poor differentiation and lack of cohesion. The intestinal and diffuse types are pathologically considered as separate entities. When a tumor does not fit into these two types, it is classified as mixed [45]. The histological type was determined in all cases based on the pathological report of the resection specimen.

### 4.3. HIPEC

Indication for the additional use of HIPEC was carried out by the multidisciplinary team, based on a careful assessment of the patients’ symptoms and general condition, results of endoscopy, imaging studies (CT scan), pathology subtyping, and staging laparoscopy (unless an exploratory laparotomy has been performed elsewhere). Conversion surgery, including extended gastrectomy, at least D2 lymphadenectomy, and peritonectomy (complete cytoreductive surgery) followed by HIPEC, was performed after systemic chemotherapy for downstaged patients with oligometastatic peritoneal disease, as described previously [36].

### 4.4. Esophagojejunostomy Leak

There is no universally accepted definition of EJL. The one proposed by the UK Surgical Infection Study Group (SISG) in 1991 has not been widely adopted [46]. In this study, postoperative EJL was diagnosed when the patient presented with fever, abdominal pain, leukocytosis, and elevation of C-reactive protein (CRP). The EJL was confirmed by the discovery of drain discharge, during endoscopy examination or by performing water-soluble passage graph or computed tomography (CT) with oral contrast.

### 4.5. Morbidity Evaluation

The Clavien–Dindo classification [47,48] and Comprehensive Complication Index (CCI) [49] scales were used to assess postoperative complications. Complications were prospectively recorded and classified according to the list of the Gastrectomy Complications Consensus Group [50].

### 4.6. Statistical Analysis

All analyses were performed using MedCalc 15.8 (MedCalc Software, Ostend, Belgium). Data were expressed as a percentage (for the categorized variable), mean, standard deviation, median, and range (for continuous variables). We considered *p* values below 0.05 (two-sided) to be statistically significant. The Spearman correlation test was used to calculate the correlation coefficients. The evaluation of the EJL and postoperative complications according to demographic and clinical (categorized) variables was performed using odds ratio test (multivariate analysis was performed with the use of logistic regression method). The comparison of the values of selected indicators depending on selected demographic and clinical variables was carried out using non-parametric tests (data had a different distribution than normal): ANOVA Kruskal–Wallis (for more than 2 groups) or U-Mann–Whitney (for 2 groups). Overall survival (OS) was defined as the length of time from the date of gastrectomy to the patient’s death (complete data) or last documented follow-up (censored data). Univariate OS analysis was performed with the use of the Kaplan–Meier estimation method (log-rank), whereas Cox logistic regression models were used in multivariate OS analyses, with statistically significant factors from univariate analysis (α < 0.05) as included variables.

## 5. Conclusions

The mixed histological type of GC is a new, tumor-related risk factor for EJL. HIPEC was confirmed to be a risk factor for postoperative complications after gastrectomy.

## Figures and Tables

**Table 1:** Clinicopathological variables of all patients included in the study.

Variable	No. of Patients *n* = 114 (%)
Sex	
Male	67 (58.8%)
Female	47 (41.2%)
Age (years)	
Average	57.9
Standard deviation (±)	12.5
Median (min-max)	58 (28–80)
Lauren histological type	
Intestinal	42 (37.0%)
Mixed	33 (28.7%)
Diffuse	39 (34.3%)
pT	
T0	5 (4.5%)
T1a	1 (0.9%)
T1b	6 (5.4%)
T2	17 (15.3%)
T3	48 (42.3%)
T4a	23 (19.8%)
T4b	14 (11.7%)
pN	
N0	46 (40.9%)
N1	14 (11.8%)
N2	20 (17.3%)
N3a	23 (20.0%)
N3b	11 (10.0%)
pM	
M0	84 (73.7%)
M1	30 (26.3%)
Grading	
G1	7 (3.9%)
G2	37 (32.4%)
G3	69 (63.7%)
Neoadjuvant chemotherapy	
Yes	81 (71.1%)
No	33 (28.9%)
HIPEC	
Yes	22 (19.3%)
No	92 (80.7%)
Surgical margin	
R0	105 (92.2%)
R1	9 (7.8%)
Surgical margin (mm)	
Average	26
Standard deviation (±)	23
Median (min-max)	20 (5–100)
Reconstruction method	
TG (Roux-en-Y)	98 (86.0%)
PG+DTR	16 (14.0%)
Postoperative EJL leak	
Yes	5 (4.5%)
No	109 (95.5%)
Postoperative complications	
Yes	46 (40.4%)
No	68 (59.6%)
CCI	
Average	17.4
Standard deviation (±)	26.1
Median (min-max)	0 (0–100)
Hospitalization time	
Average	12.9
Standard deviation (±)	8.2
Median (min-max)	11 (4–59)
ICU hospitalization time	
Average	6.9
Standard deviation (±)	5.1
Median (min-max)	5 (1–20)

HIPEC—hyperthermic intraperitonal chemotherapy, EJL—esophagojejunostomy leak, CCI—comprehensive complication index, ICU—intensive care unit, TG—total gastrectomy, PG—proximal gastrectomy, DTR—double tract reconstruction.

**Table 2:** Univariate and multivariate analysis of the risk of EJL and postoperative complications.

Variable	EJL *n* (%)		Univariate	Multivariate	Postoperative Complications *n* (%)		Univariate	Multivariate
	Yes	No	OR (95%CI) *p*	OR (95%CI) *p*	Yes	No	OR (95%CI) *p*	OR (95%CI) *p*
Sex			0.12	0.94			1.00	1.30
Male	5 (100%)	60 (56.07%)	(0.01–2.15)	(0.41–2.15)	27 (58.7%)	40 (58.8%)	(0.47–2.15)	(0.54–3.12)
Female	0 (0%)	49 (43.92%)	0.1478	0.8894	19 (41.3%)	28 (41.2%)	0.9891	0.5595
Age			0.25	0.79			1.16	1.07
<58 years	4 (80%)	53 (49.53%)	(0.03–2.27)	(0.45–1.36)	24 (52.2%)	33 (48.5%)	(0.55–2.45)	(0.46–2.46)
≥58 years	1 (20%)	54 (50.47%)	0.2156	0.3919	22 (47.8%)	35 (51.5%)	0.7027	0.8735
Lauren type			**11.11**	**12.45**			0.81	0.88
Mixed	4 (80%)	27 (26.47%)	**(1.19–103.86)**	**(1.03–150.10)**	14 (31.1%)	17 (27.0%)	(0.35–1.90)	(0.34–2.81)
Intestinal, Diffuse	1 (20%)	75 (73.53%)	**0.0347**	**0.0472**	31 (68.9%)	46 (73.0%)	0.6404	0.7862
Lauren type			0.17	0.81			0.77	1.62
Diffuse	0 (0%)	36 (35.29%)	(0.01–3.08)	(0.71–3.48)	17 (37.8%)	20 (31.7%)	(0.34–1.71)	(0.65–3.99)
Intestinal, Mixed	5 (100%)	66 (64.71%)	0.2280	0.2634	28 (62.2%)	43 (68.3%)	0.5153	0.3011
Lauren type			0.40	0.99			1.56	0.71
Intestinal	1 (20%)	39 (38.23%)	(0.04–3.75)	(0.70–3.42)	14 (31.1%)	26 (41.3%)	(0.69–3.48)	(0.29–1.73)
Mixed, Diffuse	4 (80%)	63 (61.76%)	0.4250	0.2811	31 (68.9%)	37 (58.7%)	0.2824	0.4529
Grading			2.78	1.46	15 (35.7%)	22 (36.7%)	0.96	1.10
G1, G2,	3 (60%)	34 (35.05%)	(0.44–17.45)	(0.81–4.01)	27 (64.3%)	38 (63.3 %)	(0.42–2.18)	(0.44–2.78)
G3	2 (40%)	63 (64.95%)	0.2755	0.1484	15 (35.7%)	22 (36.7%)	0.9216	0.8344
pT	3 (60%)	72 (68.57%)	0.69	0.78	30 (65.2%)	46 (70.8%)	0.77	1.06
T0, T1a, T1b, T2, T3 T4a, T4b	2 (40%)	33 (31.43%)	(0.11–4.31)	(0.08–7.53)	16 (34.8%)	19 (29.2%)	(0.34–1.74)	(0.43–2.63)
			0.6892	0.8285			0.5356	0.8983
pN			0.35	0.42			0.88	0.93
N0	1 (20%)	43 (41.35%)	(0.04–3.28)	(0.04–4.10)	18 (39.1%)	27 (42.2%)	(0.41–1.91)	(0.39–2.18)
N1a, N2, N3a, N3b	4 (80%)	61 (58.65%)	0.3613	0.4532	28 (60.9%)	37 (57.8%)	0.7478	0.8631
pM			1.49	1.42	32 (69.6%)		0.70	0.85
M0	4 (80%)	78 (72.90%)	(0.16–13.86)	(0.11–18.61)	14 (30.4%)	52 (76.5%)	(0.30–1.63)	(0.27–2.43)
M1	1 (20%)	29 (27.10%)	0.7275	0.7918	32 (69.6%)	16 (23.5%)	0.4124	0.7142
Surgical margin			1.44	1.11			0.94	0.87
R0	5 (100%)	98 (91.6%)	(0.07–27.63)	(0.11–11.59)	41 (%)	61 (%)	(0.27–3.17)	(0.24–3.11)
R1	–	9 (8.4%)	0.8089	0.9308	5 (%)	7 (%)	0.9218	0.8329
No. of removed lymph nodes			10.79	14.53			1.70	1.44
Yes	5 (100%)	52 (50.5%)	(0.58–200.17)	(1.47–143.73)	27 (60%)	30 (46.9%)	(0.78–3.68)	(0.65–3.22)
No	-	51 (49.5%)	0.1104	0.9939	18 (40%)	34 (53.1%)	0.1782	0.3673
HIPEC			1.02	2.40			**0.30**	**3.88**
Yes	1 (20%)	21 (19.63%)	(0.11–9.64)	(0.18–31.86)	32 (69.6%)	60 (88.2%)	**(0.16–0.80)**	**(1.40–10.80)**
No	4 (80%)	86 (80.37%)	0.9836	0.5074	14 (30.4%)	8 (11.8%)	**0.0162**	**0.0094**
Reonstruction method			1.71	1.75			0.85	1.39
TG (Roux-en-Y)	5 (100%)	93 (86.92%)	(0.09–32.51)	(0.26–11.63)	39 (84.8%)	59 (86.8%)	(0.29–2.47)	(0.41–4.73)
PG+DTR	0 (0%)	14 (13.08%)	0.7225	0.5612	7 (15.2%)	9 (13.2%)	0.7652	0.5969
Neoadjuvant chemotherapy			4.74	4.53			0.79	1.03
No	0 (0%)	32 (29.91%)	(0.25–88.16)	(0.05–5.06)	12 (26.1%)	21 (30.9%)	(0.34–1.82)	(0.39–2.66)
Yes	5 (100%)	75 (70.09%)	0.2973	0.1963	34 (73.9%)	47 (69.1%)	0.5800	0.9516

OR—odds ratio, 95%CI—95% confidence interval, n/a—not applicable, EJL—esophagojejunostomy leak; HIPEC—hyperthermic intraperitonal chemotherapy, TG—total gastrectomy, PG—proximal gastrectomy, DTR—double tract reconstruction. The statistically significant results are marked in bold.

**Table 3:** Comparison of CCI values depending on demographic, clinical and pathological factors.

Variable	CCI *Me* (25–75th Percentile)	*p*
Sex		
Male	0 (0–20.9)	0.4341
Female	0 (0–33.7)	
Age		
<58	0 (0–29.6)	0.3485
≥58	0 (0–29.6)	
Lauren type		
Intestinal	0 (0–20.9)	
Mixed	10.4 (0–50.7)	0.2172
Diffuse	0 (0–25.2)	
Grading		
G1	20.9 (5.2–20.9)	
G2	0 (0–37.0)	0.9458
G3	0 (0–29.6)	
Neoadjuvant chemotherapy		
Yes	0 (0–29.6)	0.5469
No	0 (0–29.6)	
pT		
T0	0 (0–10.6)	
T1a	0 (0–0)	
T1b	27.3 (0–68.8)	
T2	20.9 (0–20.9)	0.1846
T3	0 (0–20.9)	
T4a	0 (0–20.9)	
T4b	39.7 (0–52.3)	
pN		
N0	0 (0–20.9)	
N1a	10.4 (0–29.6)	
N2	0 (0–35)	0.8275
N3a	0 (0–35.9)	
N3b	20.9 (0–52.7)	
pM		
M0	0 (0–20.9)	0.1820
M1	0 (0–44.7)	
Surgical margin		
R0	0 (0–25)	0.6758
R1	0 (0–41)	
Reconstruction method		
TG (Roux-en-Y)	0 (0–29.60)	0.7206
PG+DTR	0 (0–25.20)	
HIPEC		
Yes	31.6 (0–54.2)	**0.0034**
No	0 (0–20.9)	
Postoperative EJL		
Yes	79.5 (58.2–100)	**<0.0001**
No	0 (0.00–20.9)	

CCI—comprehensive complication index, *Me*—median, EJL—esophagojejunostomy leak HIPEC—hyperthermic intraperitonal chemotherapy, TG—total gastrectomy, PG—proximal gastrectomy, DTR—double tract reconstruction. The statistically significant results are marked in bold.
